# Coupling sparse Cox models with clustering of longitudinal transcriptomics data for trauma prognosis

**DOI:** 10.1186/s13040-021-00257-8

**Published:** 2021-04-14

**Authors:** Cláudia S. Constantino, Alexandra M. Carvalho, Susana Vinga

**Affiliations:** 1 INESC-ID, Instituto Superior Técnico, ULisboa, R. Alves Redol 9, Lisbon, 1000-029 Portugal; 2 Instituto de Telecomunicações, Instituto Superior Técnico, ULisboa, Av. Rovisco Pais 1, Lisbon, 1049-001 Portugal; 3 IDMEC, Instituto Superior Técnico, ULisboa, Av. Rovisco Pais 1, Lisbon, 1049-001 Portugal

**Keywords:** Longitudinal gene expression data, Regularised optimisation, Pattern mining, Imputation, Multivariate time series clustering

## Abstract

**Background:**

Longitudinal gene expression analysis and survival modeling have been proved to add valuable biological and clinical knowledge. This study proposes a novel framework to discover gene signatures and patterns in a high-dimensional time series transcriptomics data and to assess their association with hospital length of stay.

**Methods:**

We investigated a longitudinal and high-dimensional gene expression dataset from 168 blunt-force trauma patients followed during the first 28 days after injury. To model the length of stay, an initial dimensionality reduction step was performed by applying Cox regression with elastic net regularization using gene expression data from the first hospitalization days. Also, a novel methodology to impute missing values to the genes selected previously was proposed. We then applied multivariate time series (MTS) clustering to analyse gene expression over time and to stratify patients with similar trajectories. The validation of the patients’ partitions obtained by MTS clustering was performed using Kaplan-Meier curves and log-rank tests.

**Results:**

We were able to unravel 22 genes strongly associated with hospital’s discharge. Their expression values in the first days after trauma showed to be good predictors of the length of stay. The proposed mixed imputation method allowed to achieve a complete dataset of short time series with a minimum loss of information for the 28 days of follow-up. MTS clustering enabled to group patients with similar genes trajectories and, notably, with similar discharge days from the hospital. Patients within each cluster have comparable genes’ trajectories and may have an analogous response to injury.

**Conclusion:**

The proposed framework was able to tackle the joint analysis of time-to-event information with longitudinal multivariate high-dimensional data. The application to length of stay and transcriptomics data revealed a strong relationship between gene expression trajectory and patients’ recovery, which may improve trauma patient’s management by healthcare systems. The proposed methodology can be easily adapted to other medical data, towards more effective clinical decision support systems for health applications.

## Introduction

Temporal data has been frequently used in medical research to follow disease progression over a few time points or prolonged periods. Hereupon, longitudinal studies help understanding patterns of change over time, and its analysis is receiving increasing attention [1]. For instance, multivariate time series and longitudinal data collected from a hospital information system of a medical center aided providing higher quality medical services to patients through a better management strategy and decision-making [2]. Also, time-series gene expression data have shown to be important in discovering complex biological interactions and clinical mechanisms [3]. Unfortunately, the number of genetic markers measured in these types of studies, which has ranged from thousands of genes to millions of genetic variants, leads to significant computational challenges, specially when analysing temporal omics data.

In high-dimensional omics data, the number of variables (genes) measured in these experiments is usually much higher than the sample size (number of subjects included in the study). From a statistical point of view, this can be a nuisance due to associated identifiability problems of the parameter estimation procedure. Therefore, identifying accurate and relevant biomarkers in a high-dimensional data set has become one of the key challenges today for the advance of precision medicine.

Different statistical methods have been proposed to deal with high-throughput data analysis, which is essential for an effective and reproducible variable selection, including software available for the storage and retrieval of large data sets, data mining in transcriptomics, and integrative interactomics [4–6]. The application of these methods is significantly broad, and includes oncological and other clinical research [7, 8]. In particular, the availability of high-dimensional longitudinal data has been applied in trauma studies, given the socio-economic impact of the associated pathologies.

Traumatic diseases are a significant public health concern being the number one cause of death in young adults aged 1–44 years old and is expected to rise to the leading cause of death in older age groups [9]. Trauma is related to a severe physical injury caused by an accident or violence, which may result in infections, sepsis [10], and multiple organ failure (MOF) [11], to name just a few pathologies. Traumatic diseases constitute a significant and worrying cause of disability, long-term morbidity, suffering, and healthcare resource consumption [12, 13]. Therefore, trauma disease is an authentic challenge for the healthcare system, and the improvement of patient’s management by the healthcare systems may contribute to reduce mortality and improve the functional condition and life quality of the survivors [14].

To improve healthcare systems in treating severe systemic inflammation and understanding its key regulatory elements and molecular signatures, the National Institute of General Medical Sciences supported the “Inflammation and the Host Response to Injury” (IHRI) research program. One of the IHRI large-scale outcomes is the Trauma-Related Database (TRDB), containing a 28-day prospective clinical genomics study involving a cohort of 168 patients, ∼800 microarrays, and a set of clinical variables. Thereby, a high-dimensional time-series gene expression data analysis is of paramount importance for improving hospital management, facilitating decision-making, and thus advance personalised medicine.

Our study is based on the data provided by IHRI program (available at http://www.gluegrant.org upon user registration). Our proposed method aims at predicting the distribution of times until hospital discharge, by using a constellation of gene expression variables measured at multiple time points within the first 28 days after injury. Combining bioinformatics and statistical tools, we unveiled relevant gene signatures associated with patients’ recovery. Also, we studied the gene expression trajectory within each subject to reveal patients’ clusters and thus predict discharge of future trauma patients by using the estimated cohort’s stratification.

Our methodology provides a novel framework for time-series gene expression profiling to elucidate trauma patient prognosis after injury, that can be easily extended to many other applications that model associations between follow-up times and high-dimensional multivariate time-series (MTS).

## Material and methods

**Fig. 1:**
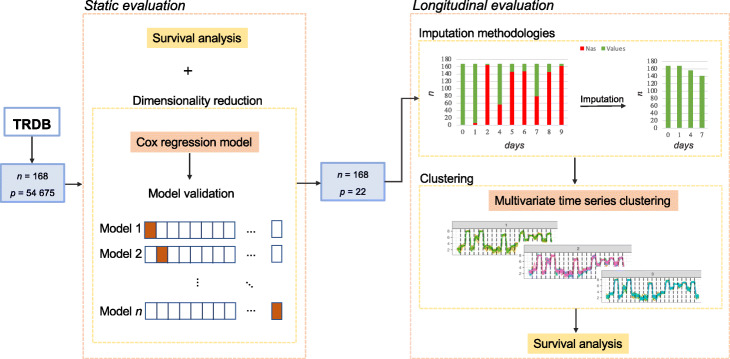
Schematic representation of the framework used in the study. Firstly, a static evaluation was performed for specific time points from the TRDB. This step includes a survival analysis based on the patient discharge from the hospital and dimensionality reduction of variables using Cox regression models. Secondly, a longitudinal evaluation for the entire time-series gene expression data of trauma patients was done. Imputation methodologies were implemented, and multivariate time series clustering was applied to profile trauma patients

This section presents the data under study and the techniques used to reduce the dimensionality of gene expression data. Since the data under study is approached from the survival perspective, a brief introduction to this subject is also given. Next, the methods used for missing data imputation and for multivariate time series clustering are explained. The overall procedure is illustrated in Fig. 1.

### Longitudinal gene expression data

The dataset under study is constituted by leukocyte gene expression with 797 microarrays from 168 blunt-force trauma patients followed in the first 28 days after they experienced an injury. Each microarray contains information about 54675 genes. The cohort of trauma patients (107 males and 61 females), aged between 16 to 55 y.o., are from a larger epidemiological study (epidemiological study: ClinicalTrials.gov identifier: NCT00257231, https://clinicaltrials.gov/ct2/show/NCT00257231), involving severe blunt-force trauma patients treated in seven U.S. Level I trauma centers across the U.S.A., from 2003 to 2011. The study was approved by the institutional review board of each center, and written informed consent was obtained from all patients. A standardised patient care protocol was applied in each hospital center to minimise the impact of possible variability. To ensure confidentially, patients were de-identified as defined by the Health Insurance Portability and Accountability Act of 1996.

The 168 patients in the prospective clinical genomics study consented for blood sampling and with mRNA quality and array quality satisfied. Also, they were at risk of developing MOF, infectious complications, and death. Exclusion criteria were patients with isolated traumatic brain injury. According to the original study design and without practitioners’ influence, the blood samples were collected at fixed time points up to 28 hospital days post-injury, avoiding bias in the sample collection process. The longitudinal gene expression was collected for total blood leukocytes isolated from peripheral blood samples. Among the clinical variables, the discharge day from the hospital since injury is also available and constitutes the primary outcome variable used in our survival models.

In our case study, we will consider the number of patients as *n*=168,*S*={0,1,2,3,4,5,6,7,8,9,11,13,14,15,16,20,21,22,23,27,28,29} the set of original time points, and *p*=54675, the number of variables (genes).

All the data are freely available at http://www.gluegrant.org. These data were previously analysed by Desai et al. [15].

### Survival analysis and the Cox regression model

Survival analysis studies the time until an event of interest occurs and is applied in various fields of science, especially in the medical research area. It is a set of statistical approaches to investigate the time it takes for an event of interest happens, as death, the development of a disease or, in the present case, the time since the injury occurred until patient discharge from the hospital, i.e., the length of stay. However, the event time may not be observed for some subjects within the study period, creating censored observations.

To model this type of data, the Cox regression model is a widely used method due to its flexibility and its capability of handling censored data [16, 17]. It is a semi-parametric regression model, where the hazard function *h*(*t*) at time *t* for the *i*-th patient is given by:

1 $$ h(t;\mathbf{x}_{i}) = h_{0}(t) \exp\left(\mathbf{x}_{i}^{T} \boldsymbol{\beta}\right),  $$

where ***β*** represents the unknown regression coefficients, *h*_0_(*t*) represents the baseline hazards, and **x**_*i*_=(*x*_*i*1_,…,*x*_*ip*_)^*T*^ are the covariates related to individual *i* (and where the specific sampling time of each **x**_*i*_ is omitted). In our case, this vector represents all the gene expression values for the individual *i*.

The semi-parametric likelihood function, where *h*_0_(*t*) is not specified and which is related to the Cox regression model, is given by:

2 $$ L(\boldsymbol{\beta})=\prod_{i=1}^{n}\left[ \frac{\exp(\mathbf{x}_{i}^{T}\boldsymbol{\beta})}{\sum_{j \in R(t_{i})}\exp\left(\mathbf{x}_{j}^{T}\boldsymbol{\beta}\right)}\right]^{\delta_{i}},   $$

where *δ*_*i*_ is an indicator function of the censored observations, and where *R*(*t*)={*j*:*t*_*j*_≥*t*} denotes the set of all individuals that are at risk at time *t*, i.e., with a follow-up time greater than or equal to *t*.

Then, the unknown regression coefficients, ***β***, are calculated with the maximisation of the partial log-likelihood function, as follows:

3 $$ l(\boldsymbol{\beta}) = \sum_{i=1}^{n} \delta_{i} \left(\mathbf{x}_{i}^{T} \boldsymbol{\beta} - \log \sum_{j \in R(t_{i})} \exp \left(\mathbf{x}_{j}^{T} \boldsymbol{\beta} \right) \right).   $$

As proposed by [18], the estimators for the baseline hazard, *h*_0_(*t*_*i*_), can be obtained by:

4 $$ \hat{h}_{0}(t_{i})=\frac{1}{\sum_{j_{\in} R(t_{i})}\exp\left(\mathbf{x}_{j}^{T}\boldsymbol{\beta}\right)}.   $$

Thus, the partial log-likelihood in Eq. (3) for the Cox regression model is the following:

5 $$ l(\boldsymbol{\beta},h_{0})=\sum_{i=1}^{n} -\exp \left(\mathbf{x}_{i}^{T}\boldsymbol{\beta}\right)H_{0}(t_{i})+\delta_{i} \left[ \log (h_{0}(t_{i}))+\mathbf{x}_{i}^{T}\boldsymbol{\beta} \right],   $$

where *H*_0_(*t*_*i*_) is the cumulative baseline hazard function.

#### Elastic net regularisation method

To deal with high-dimensional datasets, where the number of variables (genes) is much higher than the number of observations (patients), (*p*≫*n*), a dimensionality reduction step is fundamental. In the literature, there are several techniques for variable selection to reduce the dimensionality, thus providing a sparse estimate of ***β***. In this context, elastic net has become a classical regularisation method that limits the solution space by imposing sparsity and small coefficients for the parameters, combining *ℓ*_1_-norm (sum of the absolute values of the coefficients) and *ℓ*_2_-norm (sum of the squared error of the coefficients) [19]. The balance between sparsity and the correlation among variables gives high flexibility for different types of datasets and regression models. For instance, it penalises the partial log-likelihood function (5) in the Cox’s regression model for survival. Thus, Eq. (5) becomes:

6 $$ l(\boldsymbol{\beta}) = \sum_{i=1}^{n} \delta_{i} \left(\mathbf{x}_{i}^{T} \boldsymbol{\beta} - \log \sum_{j \in R(t_{i})} \exp \left(\mathbf{x}_{j}^{T} \boldsymbol{\beta} \right) \right) - \lambda \Psi(\boldsymbol{\beta})   $$

with

7 $$ \Psi(\boldsymbol{\beta}) = \alpha \left\lVert \boldsymbol{\beta} \right\rVert_{1} + (1-\alpha) \left\lVert \boldsymbol{\beta} \right\rVert_{2}^{2},   $$

where *λ* controls the penalisation weights and *α*,0≤*α*≤1, is a controller between *ℓ*_1_ and *ℓ*_2_ penalties, given a fixed *λ*. Particularly, if *α* = 0, the ridge regression [20] is obtained. On the other hand, if *α* = 1, we are dealing with the Lasso (least absolute shrinkage and selection operator) regression [21].

The elastic net adapted for Cox’s regression can be considered as an automatic implementation of best-subset selection in some asymptotic sense. This approach simultaneously selects significant variables and estimate regression coefficients. The asymptotic properties of the Lasso-type estimator, which could be analogously derived for the elastic net adapted to Cox’s regression can be seen in [22, 23]. According to Fan and Li [24], there are three important properties a good penalty function must have: sparsity, continuity, and unbiasedness. If an estimation procedure is consistent for variable selection and yields estimators with asymptotic normality, then it possesses oracle properties, which are crucial for evaluating its merits. Elastic net adapted for Cox’s regression enjoys all the established oracle properties exhibiting variable selection consistency and asymptotic estimation normality as required (for more details, see [25]).

### Missing data imputation

Missing data are ubiquitous in clinical studies, leading to difficulties in subsequent statistical analysis, specially when dealing with longitudinal patient data [26, 27]. The presence of incomplete information constitutes a considerable challenge both in the analysis and interpretation of results and can influence the conclusions validity.

Longitudinal clinical data with missing values may occur for multiple reasons, such as failure to attend medical appointments, or lack of measurements in a particular visit. Another problem is that sometimes the intervals between sample points are not evenly spaced for a given patient, and often are different between patients, i.e., the observations are not synchronised. To correctly model the longitudinal data for all the cohort, one must take these issues into account and try to uniformize as much as possible the information. Particularly in this 28-days prospective transcriptomics study, missing values appear because blood samples were collected in different time points within the 28 days after injury across patients. Blood samples were mainly collected at specific time points, although with some variability due to daily clinical constraints. Moreover, some patients were discharged before the 28th day, leading to a loss in the gene expression follow-up after that time point.

A variety of methods were developed to complete the information for the missing time points, which often lead to good results, including the following time series imputation strategies applied in the present study: (i) omitting the incomplete entries; (ii) imputation based on the Last Observation Carried Forward (LOCF); (iii) linear interpolation; and (iv) present proposal which combines imputation methodologies to apply in time-series gene expression data.

The first approach is a basic strategy and corresponds to omitting the missing values, leading to a different trajectory evaluation and sampling scheme for each cohort patient.

In the second, missing values are replaced with the last known observation of the same patient, i.e., the last observation is carried forward [28].

The third method uses linear interpolation to replace missing values. It assumes a linear relationship between the missing and non-missing values, i.e., using the non-missing values from adjacent data points to compute a missing data point. Linear interpolation has already been shown to have overall superiority in replacing missing values than other complex techniques [29].

**Fig. 2:**
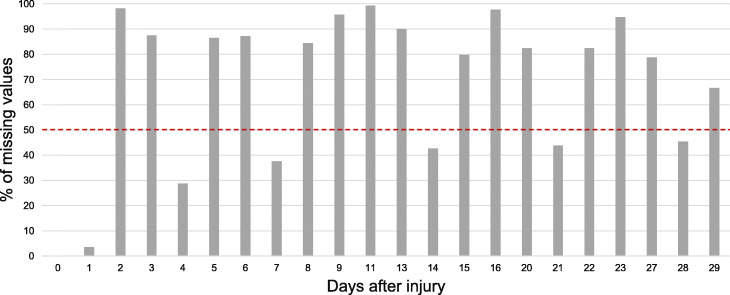
Inspection of missing data by days. Percentage of missing values in the set of original time points, i.e., the set of days with gene expression measures. The time points with less than 50% of missing values are *T*={0,1,4,7,14,21,28}

Finally, to achieve the most reliable imputation for a time-series gene expression data within a range of days, like the one used in our study, a combination of these three methodologies (i)–(iii) is proposed and presented in the Algorithm 1. For the input, in addition to the original data, it is necessary to define the time points *T* to be processed and maintained to the algorithm output. In our study, we obtain *T*, being *T*⊂*S*, calculated such that the percentage of missing data in each time point is less than 50% (*T*={0,1,4,7,14,21,28}), as presented in Fig. 2. In future studies, this can be added as a parameter of the procedure in order to analyse the impact of different levels of missingness.

**Fig. 3:**
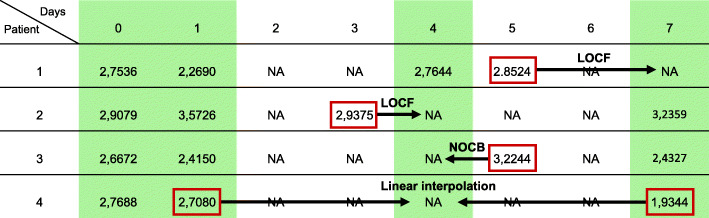
Example of the imputation methods applied in the study. Four examples of how Last Observation Carried Forward (LOCF), Next Observation Carried Backwards (NOCB), and linear interpolation are performed (Algorithm 1). The selected time points $T=\{T_{q}\}_{q=1}^{Q}$ are shaded in green and the arrows illustrate the imputation procedure


To utilise the most of the available original data, the nearest measurement was carried to selected time points *T*, but only when that difference did not exceed two days. Besides LOCF, we implemented a similar strategy named Next Observation Carried Backwards (NOCB). NOCB replaces missing values by the next known observation of the same patient. This strategy is particularly useful when the closest observation of the required missing value actually occurs very near in the future (e.g., 8th day instead of the required 7th). If both LOCF and NOCB cannot be applied (due to a large time interval up to the nearest available measurements), linear interpolation is instead performed. The approximation takes into account the closest point in the past and the closest one in the future to impute the missing value. A representative example of how the methods are applied in data is in Fig. 3. With the method implemented in Algorithm 1, the output is a dataset with fewer time points but without any missing values. Hereafter, the complete data $ \{ \mathbf {x}_{i}^{T_{q}} \}_{T_{q} \in T}$ with *i*=1,…,*n* will be used for the analysis.

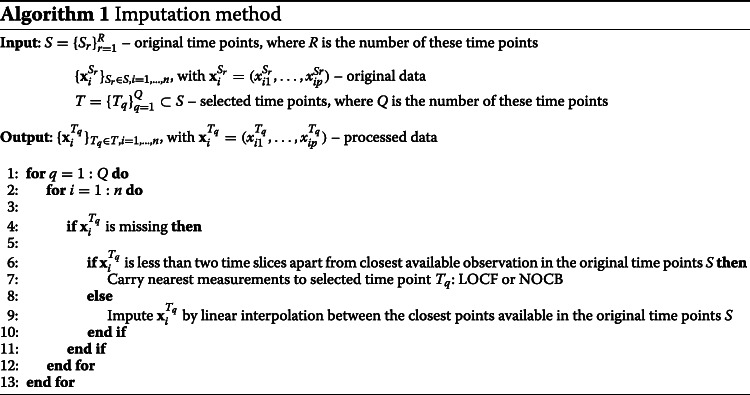


### Multivariate time series clustering

Time-series clustering has played an essential role in pattern-mining discovery and shown to provide useful information about this type of data, especially for gene expression data [30]. Numerous methods have been developed to overcome this challenging problem to analyse gene expression data [31–34]. However, most approaches rely only on univariate time series. In a multivariate framework, involving several features across each observation, multivariate time series (MTS) clustering may be fundamental to reveal groups with similar behavior. Specifically, in gene expression data, the clustering of MTS genes may unveil critical clusters of patients, with similar pattern of genes. One of the primary focus of this paper is the clustering of MTS gene expression of trauma patients within the first 28 days after injury.

Therefore, to investigate whether there are similar patterns of change among trauma patients over time, we studied similarity in time-series prioritising change patterns over time. The MTS clustering was performed using the R package dtwclust [35, 36], which is based on time series clustering along with optimisations for the dynamic time warping (DTW) distance.

Our study applied a partitional clustering, which uses by default partition around medoids (PAM) centroids. Also, we defined Global Alignment Kernels (GAK) for distance measures calculation and not the DTW distance. GAK was proposed by Cuturi et al. [37], and assesses time series similarity by using kernels. It considers the cost over all possible alignment distances that map time series onto each other as the positive kernel. The GAK distance is considered one of the most coherent measures and has been shown to provide the most stable results compared with other distance measures, as for instance DTW distance [38]. To reduce global alignment kernel’s complexity, it is used a triangular local kernel for integers where is possible to specify the kernel’s order. In the R package dtwclust, the GAK function is implemented performing normalisation and subtraction in order to obtain a distance measure that can be used in clustering procedures.

For MTS clustering, let us consider two patients, being **x**_*i*_ the multivariate time series of the *i*-th patient, and **x**_*j*_ the multivariate time series of the *j*-th patient, both with length *q*,*r*={1,2,...,7}. The first step involves creating a local cost matrix (*LCM*_*i*,*j*_) defined by:

8 $$ LCM_{ij}(q,r) = \lVert \mathbf{x}_{i}^{T_{q}} - \mathbf{x}_{j}^{T_{r}}\rVert,   $$

where $\mathbf {x}_{i}^{T_{q}}=\left (x_{i1}^{T_{q}},\ldots,x_{ip}^{T_{q}}\right), \mathbf {x}_{j}^{T_{q}}=\left (x_{j1}^{T_{r}},\ldots,x_{jp}^{T_{r}}\right)$, and *T*_*q*_ and *T*_*r*_ represent time points from *T*={0,1,4,7,14,21,28}. Note that each multivariate series has the same number of variables (genes).

Thus, a local cost matrix is created for each pair of multivariate time series (observation *ivs* observation *j*). In the second step, the cross-distance matrix is calculated using the GAK algorithm for each *LCM*_*ij*_, which interactively steps through these local cost matrices with a kernel to achieve an exponentiated soft-minimum. After the cross-distance matrix is calculated, the desired clusters for all observations included in the study are achieved.

## Results and discussion

This section reports the results obtained by the pipeline illustrated in Fig. 1. The main goal is to identify a set of genes that are associated with the time until the discharge, and also uncover relevant patient stratification, by using the methods described.

To ensure full reproducibility of our results, all the R code and data are available at https://github.com/sysbiomed/TraumaRDB.git.

### Identification of relevant genes

To unravel the most relevant genes in trauma patients to predict the discharge hospital day since the injury, an analysis of the gene expression was performed for the first days after injury (days 0, 1 and 4), taken independently. Thus, patients’ gene expression was analysed at Days 0, 1, and 4, individually, and via supervised learning.

Since the availability of this high number of genes may hamper model interpretability, sparse methods were first applied to the data. Dimensionality reduction was accomplished with elastic net regularisation by fitting the Cox regression model for survival, using all the available information regarding the time until hospital discharge, and considering the gene expression values as features. First, a preliminary analysis was accomplished to understand if it is possible to predict recovery during the first days after injury. To do so, 70% of the dataset were randomly split for training the model, and the remaining 30% for the test set. The model was obtained for the training set with the *λ* parameter estimated using cross-validation. Different *α* parameters were also tested: 0.2,0.4,0.6,0.8 and 1, in order to evaluate the impact of this choice in the results. To select the best *α*, the Harrell’s *c*-index [39], widely used as a measure of separation of two survival distributions, was calculated in the test set for each model (see Table 1). The *α* value giving the highest *c*-indexes across the three days (Day 0, Day 1, and Day 4) was 0.8. So, *α*=0.8 is the parameter value chosen for further analysis.

**Table 1:** Harrell’s *c*-indexes calculated in the test set for several model parameters *α* for Days 0, 1, and 4

	*α*				
*c*-index	0.2	0.4	0.6	0.8	1.0
Day 0	0.59	0.60	**0.69**	**0.69**	0.67
Day 1	**0.72**	0.69	0.68	**0.72**	0.66
Day 4	0.71	0.72	0.72	**0.75**	0.71

**Fig. 4:**
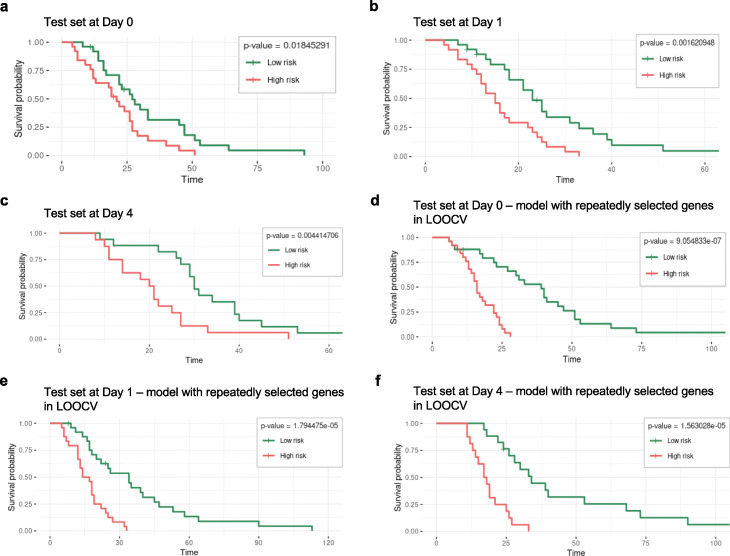
Kaplan-Meier (KM) curves for Days 0, 1, and 4. **a** - **c** KM curves from Day 0, Day 1, and Day 4 test sets (30%), respectively. **d** - **f** KM curves from Day 0, Day 1, and Day 4 test sets after LOOCV, respectively. The event of interest corresponds to patient discharge from hospital

The fitted model (*α*=0.8 and cross-validated *λ*) was then used to separate the test set into high and low-risk groups using Kaplan-Meier survival curves. In our study, high risk corresponds to patients with earlier discharge from the hospital, and low risk the inverse case. This procedure was repeated for each dataset day (Days 0, 1, and 4) and the correspondent Kaplan-Meier curves are presented in Fig. 4a to c. The difference between high and low-risk individuals was statistically significant in the test set, confirming that the biomarkers selected by this method may have good prognostic value.

For each test set presented in Fig. 4a to c, the *c*-indexes achieved were 0.69, 0.72, and 0.75, respectively for Day 0, Day 1, and Day 4. At a first analysis, it seems possible to predict hospital discharge based on the selected gene expression time profiles in these first days after injury.

Secondly, leave-one-out cross-validation (LOOCV) was performed to detect genes strongly associated with patients’ recovery. For model estimation, LOOCV considers all observations except one, which is left out from the training set. So, models are calculated the same number of times as the number of observations (*n*=168) in the dataset. As in the previous task, the same parameters and procedure were used for model prediction (*α*=0.8, cross-validated *λ*). The intersection of genes appearing in all the *n*=168 models calculated with LOOCV correspond to the genes that may be more strongly associated with patients recovery. For Days 0, 1, and 4, the following genes appeared in all the regularised models:

- **Day 0:***AA808444*, *AI421660*, *AW474434*, *BC015390*, *BG120535*;
- **Day 1:***AF279899*, *AI127598*, *AK097281*, *AW665177*, *BC022029*, *BG120535*, *NM_013450*, *NM_173529*;
- **Day 4:***AA873542*, *AB011151*, *AW574504*, *BE906233*, *BF129339*, *NM_002600*, *NM_018368*, *NM_024669*, *NM_025151*, *X65661*.

We stress that the Cox proportional hazards assumption was tested for each of the variables using the scaled Schoenfeld residuals [40]. The test was not statistically significant for any covariate, and the global test was also not statistically significant (*p*-values >0.05). Thus, we can assume that the proportional hazard assumption is fulfilled and that the fitted Cox regression models adequately describe the data.

Interestingly, some of these genes are already known to be related with the inflammation response and the proper development and functioning of the immune system, key points to be considered for wound healing process [41]. For instance, *AW474434*, with the gene symbol *TNFSF10*, is a protein coding gene that belongs to the tumor necrosis factor (TNF) ligand family, which induces apoptosis in transformed cells having a key anti-inflammatory role [42]. Also, *BG120535*, gene symbol *VNN1*, plays a significant role in the innate and adaptive immune response, and it is extensively known for its anti-inflammatory activity [43]. The *AW574504*, which corresponds to the *PECAM1* gene symbol, is another protein coding gene with important functions in cell junction and in leukocyte trafficking and immune response [44]. Another interesting example is *NM_002600*, also known as *PDE4B2* gene, which is a key regulator of many important physiological processes, specifically in the control of inflammation [45].

Once again, the Kaplan-Meier curves for high and low-risk groups were calculated for the test set after model estimation with these specific genes that were always selected (model validation: 70% train/30% test). As presented in Fig. 4d to f, the difference between the two groups continued to be statistically significant using the genes always selected by LOOCV technique.

To evaluate the performance of the models using these specific genes, the *c*-index in the test set was again calculated. Respectively for Day 0, Day 1, and Day 4, the *c*-indexes were 0.77, 0.80, and 0.84, respectively, which revealed that good model predictions were achieved.

The sparse PCA method [46], which is an unsupervised learning method that attempts to find a weight vector with only a few non-zero values, was also applied to the transcriptomic data of Day 0. Unfortunately, none of the identified genes corresponded to the ones previously selected by LOOCV using Cox regression with elastic net regularisation. Hereupon, the genes that stand out with the PCA method are probably not related to the duration of hospital stay and the event (“‘patient discharge”) used to select the genes by Cox regression models, which was the exact base of the study. For another unsupervised learning approach, functional principal component analysis (PCA), which captures the dynamics over time for feature selection, could be an alternative to be applied in further studies [47].

It is also noteworthy that, in the present study, we focused only on transcriptomic variables. However, the inclusion of other patient-specific variables, such as clinical information, may further improve model performance and should be analysed in future studies.

The union of the genes previously selected for each time point originates 22 genes strongly associated with patient recovery based on the first four days after injury. These are the genes used for further analysis, including MTS clustering.

### Longitudinal evaluation

**Fig. 5:**
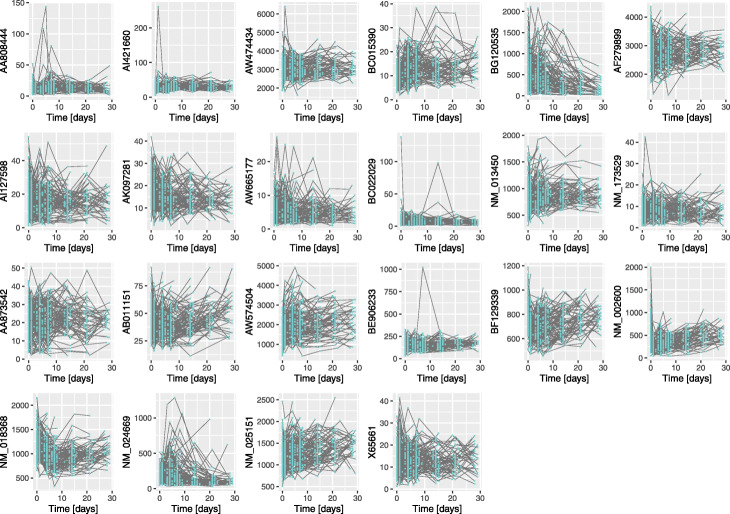
Gene expression trajectory of the 22 genes identified as relevant to predict patient discharge. Gene expression trajectory of 168 patients until the 28th day after injury. These 22 genes appear in all the models calculated with LOOCV for Day 0, Day 1, and Day 4 datasets

The analysis performed previously considers variables as static or time-independent. Henceforward, the 22 genes selected were analysed as longitudinal variables, considering blood samples collections up to the 28th day. The time trajectory of the 22 genes is illustrated in Fig. 5.

#### Imputation results

Blood samples collection were performed at fixed time points during the 28 days of follow-up. However, measurements were not always made at the same fixed time points across patients. This led to many missing values per day, and consequently made longitudinal data analysis a challenge. At this task, variables were log-transformed.

As previously described, during the 28 days of follow-up, the time points with less than 50% of missing values are represented by *T*={0,1,4,7,14,21,28}. Then, the imputation procedure described in the “Material and methods” section was applied to the corresponding observations $\{\mathbf {x}_{i}^{T_{q}}\}$, in particular by running Algorithm 1. The application of these imputation methodologies allowed to have a final dataset of shorter time series but without any missing values.

**Fig. 6:**
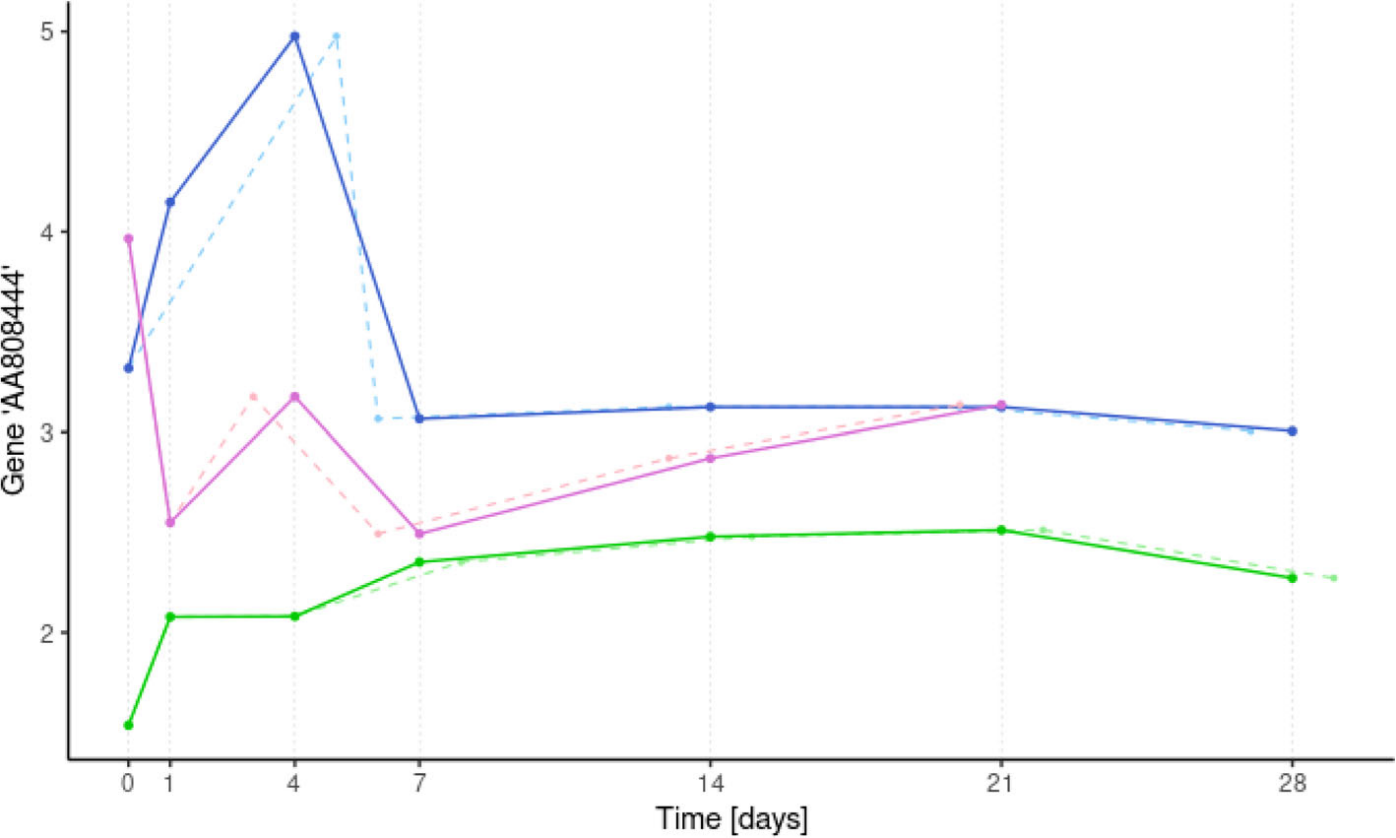
Imputation results in one gene expression of three patients randomly selected from the study. Original longitudinal gene expression data of three patients are represented by dashed lines, and the corresponding imputation results in solid lines. The trajectory of the gene *AA808444* is illustrated

In Fig. 6, an example with three patients’ trajectory randomly selected from the study is illustrated. Dashed lines represent the original gene expression data, and the corresponding imputation results are plotted in solid lines. We can observe a very close behaviour, that indeed will not compromise any of the following analysis. The full quantitative analysis of the performance of this imputation method could be estimated via simulation.

The last day of the measure would be the 28th day except in the cases where the discharge occurred earlier. There were only 33 complete cases from Day 0 to Day 28, i.e., only 33 patients were discharged from the hospital after the 28th day. 141 patients had complete gene expression information from Day 0 to Day 7, i.e., they were discharged after the 7th day in the hospital. Hereupon, the dataset was split into two independent datasets: complete cases from Day 0 to Day 28 with *n*=33 (Dataset *A*) and complete cases from Day 0 to Day 7 with *n*=141 (Dataset *B*). For both datasets the same selected 22 genes were analysed.

Another interesting approach left as a future work is to deal with these missing values as interval censoring data. Diverse methodologies have been explored for estimation and inference on the regression coefficients in the Cox proportional hazards model with interval censored data, specifically in medical studies [48, 49]. So, we propose a subsequent research study for the application of methods for interval censored data, instead of using a multiple imputation procedure to fill-in missing values.

Although sufficiently general, the data imputation procedure here described and performed should be adapted when analysing novel datasets. In fact, the optimisation of this pre-processing step is often data-dependent since it must take the particularities of each data set into account.

#### Clustering

Time-series clustering of gene expression from trauma patients may help to identify an interesting patient stratification. Each obtained cluster will include patients with similar genes patterns for the 22 genes considered. Towards this goal, MTS clustering was applied to Datasets *A* and *B* separately, by applying partitional clustering with PAM centroids and GAK distance (see “Material and methods” section).

The optimal number of clusters (*k*) for each dataset was chosen based on the *p*-value of the log-rank test comparing each cluster’s survival curves after the MTS clustering over a range of possible values for *k*. We considered the lowest *p*-value achieved to select the value of *k*. The results for both Datasets *A* and *B* are presented in Table 2. The optimal *k* chosen was 8 and 13 for Datasets *A* and *B* respectively, based on the criteria described.

**Table 2:** Log Rank and Wald test for MTS clustering over a range of *k* values

	Dataset *A* (*n*=33)		Dataset *B* (*n*=141)	
*k*	Log rank	Wald test	Log rank	Wald test
5	0.2	0.3	0.9	0.9
6	0.01	0.05	1.0	1.0
7	0.008	0.05	0.4	0.4
8	**9e-05**	**0.003**	0.4	0.4
9	3e-04	0.01	0.5	0.5
10	0.02	0.03	0.9	0.9
11 ^*^	-	-	0.07	0.1
12 ^*^	-	-	0.01	0.03
13 ^*^	-	-	**0.003**	**0.02**
14 ^*^	-	-	0.2	0.3
15 ^*^	-	-	0.1	0.2

The values in bold are the lowest *p*-values obtained in each Dataset A and B

^*^k not considered for Dataset A since it gives clusters that are too small.

**Fig. 7:**
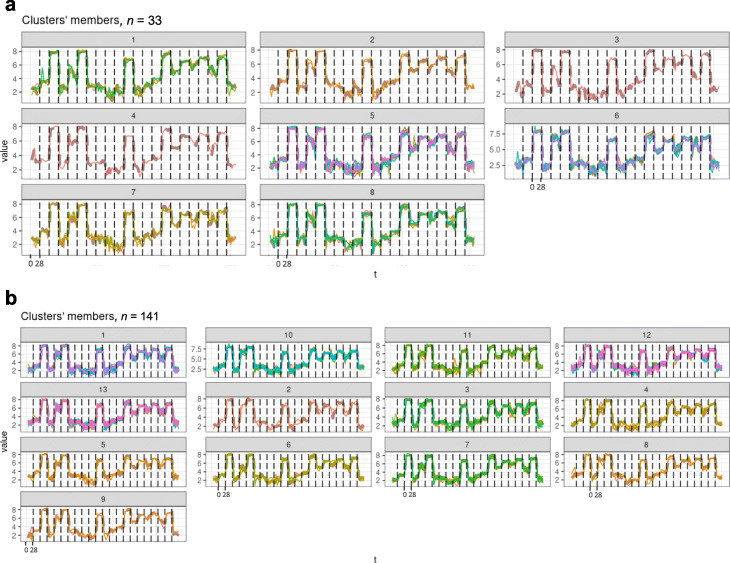
Multivariate time series clustering results for Datasets *A* and *B*. **a** MTS clusters with *k*=8 for dataset *A*. **b** MTS clusters with *k*=13 for dataset *B*. The variables (genes) are appended one after other for plotting, not for the actual clustering calculation

The result of MTS clustering with the optimal *k* values is presented in Fig. 7. Note that the variables being appended one after another is only for plotting, not for the actual clustering calculation.

**Fig. 8:**
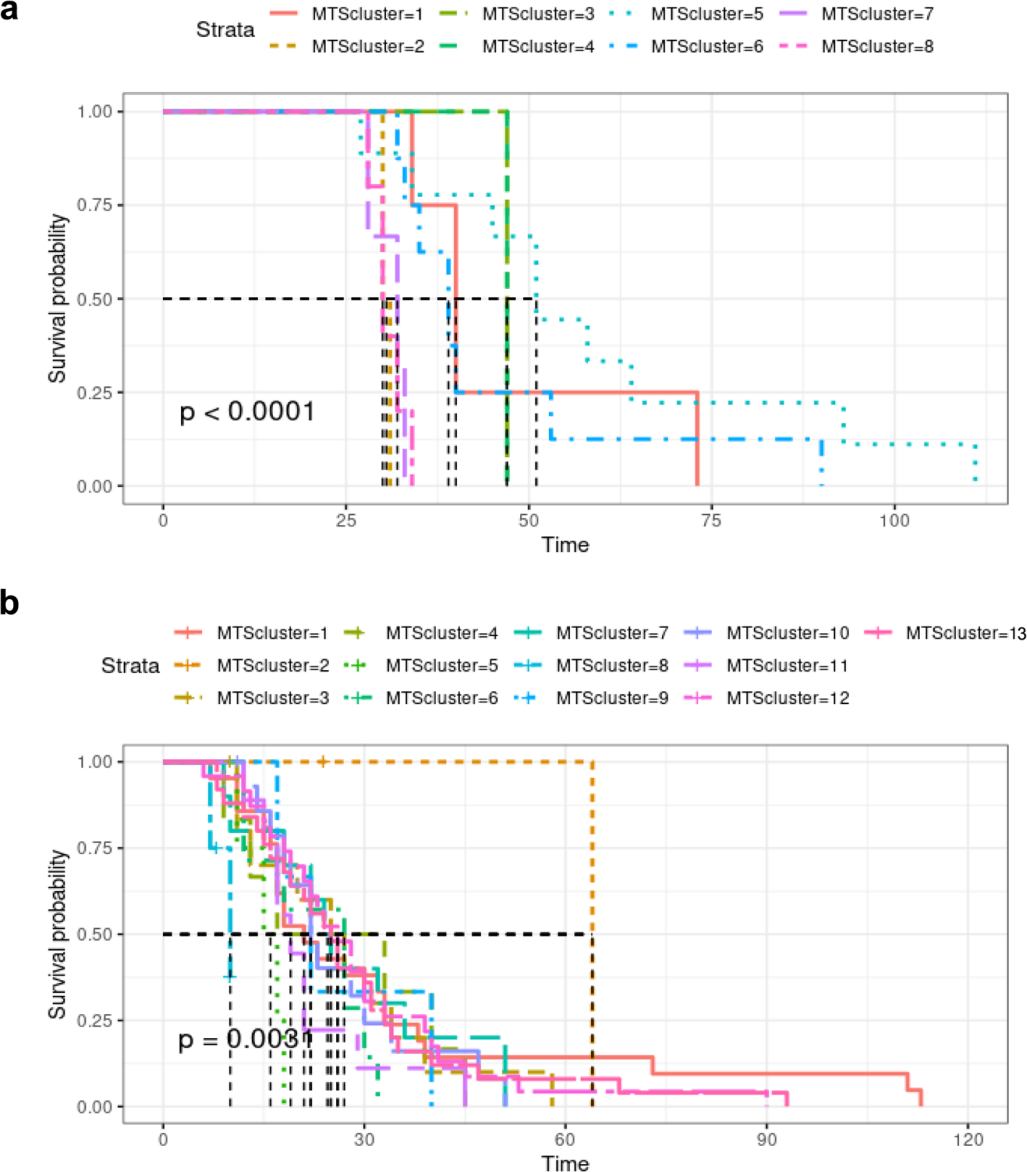
Kaplan-Meier (KM) curves resulting from the MTS clustering results. **a** KM curves obtained with MTS cluster results with *k*=8 for Dataset *A*. **b** KM curves obtained with MTS cluster results with *k*=13 for Dataset *B*

The difference between the Kaplan-Meier curves of each cluster is statistically significant for both Datasets *A* and *B*. The corresponding Kaplan-Meier graphs are presented in Fig. 8. For instance, patients in Clusters 2, 7, and 8 (Fig. 8a are discharged from the hospital in the next few days after the last follow-up (between 28th-35th day), so we may consider these as good prognosis groups. The same happens with the patients in Clusters 5 and 8 (Fig. 8b. All the patients included in these groups have similar gene expression patterns, and all were discharged early. Interestingly, the majority of patients in the good prognosis groups from Dataset *A* belong to the same strata in Dataset *B*.

Patients in the Clusters 1, 5, and 6 (Fig. 8a may be considered as the patients with the worst prognosis since they tend to be discharged later. Similarly, the Clusters 1, 12, and 13 from Dataset *B*, (Fig. 8b, are the groups of patients with worst prognosis. Once again, from the 21 patients in the groups with worst prognosis from Dataset *A* (clusters 1, 5, and 6), 19 remained placed in the same strata, in the worst prognosis groups of the Dataset *B*. Hereupon, there is a statistically significant relationship between the gene expression trajectory of these patients and their recovery, evaluated as the time until their hospital discharge.

Noteworthy, MTS clustering revealed groups of patients with similar gene expression trajectories over time, that are also associated with the severity of the disease. Indeed, the analysis of the Kaplan-Meier curves and correspoding log-rank tests for these groups revealed a strong association between the obtained stratification and the time until their hospital discharge. With these results, future patients in the healthcare system may be placed in one of the found clusters based on their gene expression over time and therefore clinicians may predict in a more accurate way when a specific patient will be discharged.

## Conclusions

Time-series gene expression data are commonly high-dimensional datasets with missing values that cannot be tackled and analysed in a straightforward way. Furthermore, the availability of time-to-event censored data, like patient follow-up information, adds an extra degree of complexity, thus requiring the longitudinal analysis to be coupled with survival models such as the Cox regression. Pattern mining discovery in this type of data, although expected to bring promising results, is still a challenge. We proposed a reliable framework to deal with a longitudinal genomic trauma-related dataset, which may provide biological and clinical insight. First, the use of regularisation techniques was able to unravel relevant genes for trauma patients’ recovery, measured as the duration of the hospital stay. A combination of imputation methodologies allowed the acquisition of a final dataset of short time series without any missing values and loss of information. Moreover, it was possible to cluster patients with similar gene expression over time, and it was noticeable a link between patients’ cluster and their discharge day from the hospital. These results may be addressed for future trauma patients entering the healthcare system, and, more generally, to improve patients’ management and further support clinical decisions.

## Data Availability

The dataset analysed during the current study and all the implemented code written with R language can be freely downloaded at github website, https://github.com/sysbiomed/TraumaRDB.git. The original study and project is available at http://www.gluegrant.org. Declarations

## Abbreviations

MOF: Multiple organ failure
IHRI: Inflammation and the host response to injury
TRDB: Trauma-related database
LOCF: Last observation carried forward
NOCB: Next observation carried backwards
MTS: Multivariate time series
PAM: Partition around medoids
GAK: Global alignment kernels
LCM: Local cost matrix
LOOCV: Leave-one-out cross-validation
